# Artificial Serum Improved

**Published:** 1908-01

**Authors:** 


Artificial Serum Improved.—Fleig maintains that the following is greatly superior to the ordinary salt solution for subcutaneous and intravenous administration. It has the power of preserving the vitality of isolated organs when immersed in it and kept at a proper temperature. Spermatozoa remain alive in it for several days:

| | | |
|---|---|---|
| ℞ | Sodium chloride .................. | 6.50 grammes |
| | Potassium chloride ................. | 0.30 gramme |
| | Magnesium sulphate ............... | 0.30 gramme |
| | Calcium cholride .................. | 0.20 gramme |
| | Sodium bicarbonate ................ | 1.0 gramme |
| | Sodium glycerophosphate ........... | 1.0 gramme |
| | Glucose ........................... | 1.0 gramme |
| | Distilled water, q. s., ad.................... | 1000 |
| | Oxygen, to saturation. | |
| M. | | |

The glucose and the oxygen are not essential and may be omitted, but are regarded as beneficial.—*La Tribune médicale.*

